# Modulation of Neurolipid Signaling and Specific Lipid Species in the Triple Transgenic Mouse Model of Alzheimer’s Disease

**DOI:** 10.3390/ijms222212256

**Published:** 2021-11-12

**Authors:** Estibaliz González de San Román, Alberto Llorente-Ovejero, Jonatan Martínez-Gardeazabal, Marta Moreno-Rodríguez, Lydia Giménez-Llort, Iván Manuel, Rafael Rodríguez-Puertas

**Affiliations:** 1 Department of Pharmacology, Faculty of Medicine and Nursing, University of the Basque Country (UPV/EHU), 48940 Leioa, Spain; estibaliz.gonzalezdesanroman@ehu.eus (E.G.d.S.R.); alberto.llorente@ehu.eus (A.L.-O.); jonatan.martinez@ehu.eus (J.M.-G.); marta.morenor@ehu.eus (M.M.-R.); ivan.manuel@ehu.eus (I.M.); 2 Department of Psychiatry and Forensic Medicine, Institute of Neuroscience, Faculty of Medicine, Universitat Autònoma de Barcelona, 08193 Bellaterra, Spain; lidia.gimenez@uab.cat; 3 Neurodegenerative Diseases, BioCruces Bizkaia Health Research Institute, 48903 Barakaldo, Spain

**Keywords:** Alzheimer’s disease, functional autoradiography, cannabinoid receptors, LPA receptors, sphingosine 1-phosphate, ligand binding, G protein, MALDI-MSI, [^35^S]GTPγS autoradiography, 3xTg-AD mice

## Abstract

Alzheimer’s disease (AD) is a progressive neurodegenerative disorder and the most common cause of dementia in aging populations. Recently, the regulation of neurolipid-mediated signaling and cerebral lipid species was shown in AD patients. The triple transgenic mouse model (3xTg-AD), harboring βAPP_Swe_, PS1_M146V_, and tau_P301L_ transgenes, mimics many critical aspects of AD neuropathology and progressively develops neuropathological markers. Thus, in the present study, 3xTg-AD mice have been used to test the involvement of the neurolipid-based signaling by endocannabinoids (eCB), lysophosphatidic acid (LPA), and sphingosine 1-phosphate (S1P) in relation to the lipid deregulation. [^35^S]GTPγS autoradiography was used in the presence of specific agonists WIN55,212-2, LPA and CYM5442, to measure the activity mediated by CB_1_, LPA_1_, and S1P_1_ G_i/0_ coupled receptors, respectively. Consecutive slides were used to analyze the relative intensities of multiple lipid species by MALDI Mass spectrometry imaging (MSI) with microscopic anatomical resolution. The quantitative analysis of the astrocyte population was performed by immunohistochemistry. CB_1_ receptor activity was decreased in the amygdala and motor cortex of 3xTg-AD mice, but LPA_1_ activity was increased in the corpus callosum, motor cortex, hippocampal CA1 area, and striatum. Conversely, S1P_1_ activity was reduced in hippocampal areas. Moreover, the observed modifications on PC, PA, SM, and PI intensities in different brain areas depend on their fatty acid composition, including decrease of polyunsaturated fatty acid (PUFA) phospholipids and increase of species containing saturated fatty acids (SFA). The regulation of some lipid species in specific brain regions together with the modulation of the eCB, LPA, and S1P signaling in 3xTg-AD mice indicate a neuroprotective adaptation to improve neurotransmission, relieve the myelination dysfunction, and to attenuate astrocyte-mediated neuroinflammation. These results could contribute to identify new therapeutic strategies based on the regulation of the lipid signaling in familial AD patients.

## 1. Introduction

The progressive and irreversible deterioration of cognitive functions present in Alzheimer’s disease (AD) include a chronic neurodegeneration characterized by pathological hallmarks including the loss of synapses, the intracellular neurofibrillary tangles (NFT) (mostly composed by hyperphosphorylated tau protein) [1], and extracellular neuritic plaques (enriched in Aβ) [2,3]. AD often has comorbidities with other severe human diseases, for example, type 2 diabetes. Some common pathophysiological alterations and signaling pathways may be involved in the association between these two disorders [4]. AD can be classified into sporadic AD, which accounts for the majority of the cases, and familial early-onset form, accounting for 1–5% of all cases, in which mutations of genes, for example, amyloid β precursor protein (APP) [5], and presenilin-1 and -2 have been suggested to underlie the development of the disease [6,7,8,9]. Aβ deposition has been related to neuroinflammatory responses, in which astrocytes and microglia play a key role [10,11]. Furthermore, the presence of NFTs has been accepted and used for *postmortem* diagnostic criteria [12]. The AD is a complex neurodegenerative disease specific to humans involving multiple factors, such as inflammation [13]. Thus, the best way to study the AD should be directly in patients. However, some animal models have been developed and must be compared to AD patients for their validation. In addition, the translational research based on “omics” technologies (including lipidomics) are increasing our knowledge of AD for the identification of early AD biomarkers [14].

The triple transgenic mouse model, 3xTg-AD, mimics many critical aspects of AD neuropathology, harboring βAPP_Swe_, PS1_M146V_, and tau_P301L_ transgenes [15,16,17,18]. These mice progressively develop the neuropathological markers of AD [19]. At 6 months of age, 3xTg-AD mice are characterized by diffuse amyloid plaques in neocortex, and intraneuronal Aβ accumulation in pyramidal neurons of the hippocampus, cortex, and amygdala [20]. Other authors have described tau phosphorylation at pyramidal layers of hippocampus [21]. This evidence suggests that tau protein dysfunction contributes to AD-related pathophysiology in 3xTg-AD mice at early ages [19]. Moreover, synaptic dysfunction and long-term potentiation deficits are already apparent at this age, although no extracellular Aβ deposits are localized at the hippocampal region. Moreover, behavioral age is an important factor in this 3xTg-AD mouse model [22,23]. Regarding the neurochemical alterations observed in AD patients, cholinergic neurotransmission seems to be one of the most characteristic alterations, including the loss of cholinergic neurons at the basal nucleus of Meynert in AD patients [24]. However, neurodegeneration is not limited to a specific neurotransmitter system; the glutamatergic [25], serotonergic [26], noradrenergic [27], and peptidergic (e.g., galanin) [28] neurotransmitter systems are also deregulated in AD [29].

Furthermore, we have recently demonstrated, in a rat lesion model of basal forebrain cholinergic neurons that shows learning and memory impairment, the specific regulation of phospholipids which is controlled by muscarinic receptor signaling [30]. Lipid molecules dynamics, finely tuned by neurotransmitter systems, may play pivotal roles in AD development. Thus, different changes occur in the composition of neural membrane glycerophospholipid, sphingolipid, and cholesterol during neurodegeneration (e.g., AD, Parkinson’s disease, and amyotrophic lateral sclerosis) [31,32,33,34,35]. These changes lead to significant increases in glycerophospholipid, sphingolipid, and cholesterol-derived lipid mediators at the plasma membrane and nuclear levels [36]. Lipid mediators are involved in neural cell proliferation, migration, cell cycle, and angiogenesis, promoting cell survival in physiological conditions [37,38,39].

As mentioned above, the study of brain lipids by lipidomic techniques, together with the analysis of neurolipid-based signaling has emerged with important neuromodulatory properties on different neurotransmitter systems in AD [30,40]. The main neurolipid systems identified so far are the endocannabinoid (eCB), and the lysophospholipid signaling systems, including the lysophosphatidic acid (LPA) and the sphingosine 1-phosphate (S1P) [41,42,43]. The endocannabinoid signaling is also modified during AD progression. Some authors suggest reduced CB_1_ densities in the entorhinal cortex, hippocampus, and caudate nucleus in advanced stages of AD patients [44]. Moreover, using immunohistochemical techniques, decreased expression of CB_1_ receptor protein was demonstrated in frontal cortex samples in AD patients [45]. Furthermore, CB_1_ receptors are up-regulated in the early stages of AD, followed by a diminution of its availability during the progress of the disease [40,46]. In contrast, other studies found no alteration of CB_1_ densities in AD brains including several cortical and hippocampal areas [47,48]. CB_1_ receptors play a fundamental role in neuroprotection, controlling excitotoxicity events related to neurodegenerative and inflammatory processes [15,49,50]. Regarding lysophospholipid systems, in vivo and in vitro studies have indicated that the neurophysiology of the LPA and S1P is relevant for AD. For example, autotaxin, an enzyme involved in LPA production, with antioxidative properties, is up-regulated in AD patients [51,52]. Moreover, LPA has been implicated in the up-regulation of BACE1 expression [53]. LPA also activates the phosphorylation of GSK-3 enzyme; LPA-mediated activation of GSK-3 occurs in the Rho pathway and may represent an important link between microtubule and microfilament dynamics in AD [54,55]. In addition, LPA has been described as a novel potential mediator in myelination [56] which is disrupted in AD [57]. Finally, the neuroprotective effects of LPA on Aβ 31–35 induced apoptosis in cultured cortical neurons have been reported [16].

The knowledge about the role of S1P-mediated signaling in AD is more recent, but indicates decreased levels of its endogenous ligand in cortical and subcortical areas in brain samples from AD patients [58] which, at least in the cortex, may be the consequence of aberrant functionality or altered expression of the S1P-synthesizing and/or degrading enzymes [59]. The S1P_1_ receptors have also been localized in astrocytes [60].

Thus, the present study analyzed the activities mediated by CB_1_, LPA_1_, and S1P_1_ receptors, respectively, in 6-month-old 3xTg-AD transgenic mice using the functional [^35^S]GTPγS autoradiography method. The receptor specificity was localized at the subcellular level by microscopic immunofluorescent localization. These results are compared to the anatomical distribution patterns of the lipid distribution in the brain of 3xTg-AD mice, obtained by MALDI mass spectrometry imaging (MALDI-MSI).

## 2. Results

### 2.1. [^35^S]GTPγS Binding Assay in 3xTg-AD Mice Brain Sections

**Figure 1:**
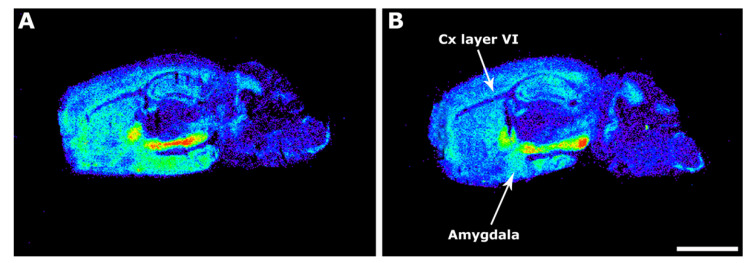
Representative autoradiograms of WT (**A**) and 3xTg-AD (**B**) mice in sagittal sections that show [^35^S]GTPγS binding stimulated by WIN55,212-2 (10^−5^ M). Note the decrease of the CB_1_–mediated activity at layer VI of the cortex (Cx Layer VI) and at the medial amygdala. Scale bar = 3 mm.

In order to assess changes between 3xTg-AD mice and age-matched wild type (WT) mice, firstly, we examined the basal G protein activity in whole brain, which was quite similar in 3xTg-AD and WT mice. The functional coupling induced by WIN55,212-2, accounting mainly for CB_1_-mediated activity, was decreased in transgenic mice compared to WT mice in the amygdala (3xTg-AD 112.8 ± 28.9% vs. WT 295.5 ± 41.7%; *p* < 0.01). This CB_1_ activity was also decreased in the VI layer of the motor cortex (3xTg-AD 238.4 ± 22.9% vs. WT 435.4 ± 58.2%; *p* < 0.01) (Figure 1, Table 1).

**Figure 2:**
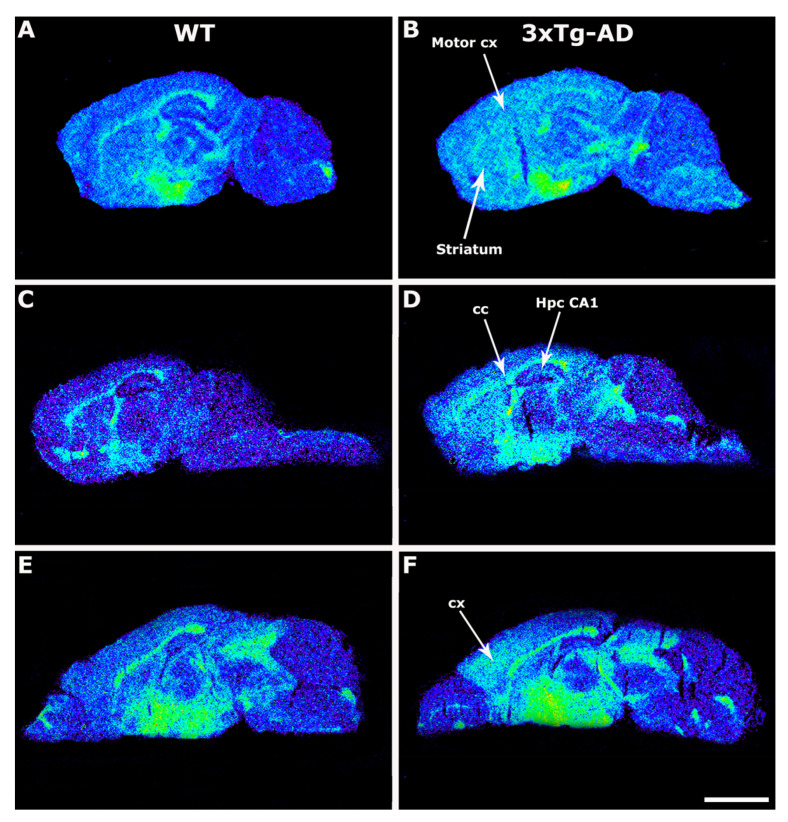
Representative autoradiograms of WT (**A**,**C**,**E**) and 3xTg-AD (**B**,**D**,**F**) mice in sagittal sections that show [^35^S]GTPγS stimulated by LPA (10^−5^ M). The [^35^S]GTPγS binding induced by LPA was increased in transgenic mice at striatum, motor cortex, corpus callosum, and hippocampal CA1 area. Scale bar = 3 mm. cc: corpus callosum, Hpc CA1: hippocampus CA1, motor cx: motor cortex, cx: cortex.

On the other hand, the [^35^S]GTPγS binding induced by LPA was increased in transgenic mice at the striatum (3xTg-AD 23.1 ± 3.8% vs. WT 3.3 ± 7.2%; *p* < 0.05), motor cortex (3xTg-AD 26.1 ± 7.6% vs. WT 6.2 ± 12.4%; *p* < 0.05), corpus callosum (3xTg-AD 189.6 ± 17.4% vs. WT 90.8 ± 12.3%; *p* < 0.05), and hippocampal CA1 area (3xTg-AD 22.7 ± 4.4% vs. WT −18.7 ± 7.8%; *p* < 0.05) (Figure 2, Table 2).

**Figure 3:**
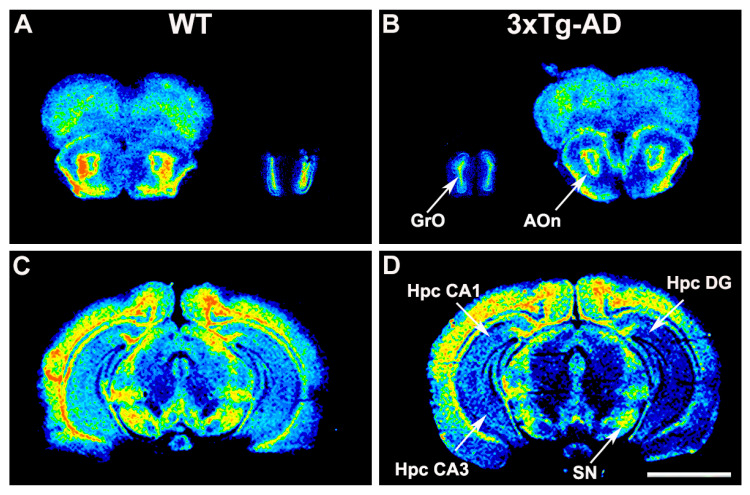
Representative autoradiograms of WT (**A**,**C**) and 3xTg-AD (**B**,**D**) mice in coronal brain sections that show [^35^S]GTPγS binding evoked by CYM5442 (10^−5^ M), accounting for S1P_1_ receptor activity. The S1P_1_ receptor activity was reduced in the rhinencephalon, hippocampus, and substantia nigra from 3xTg-AD mice. Scale bar = 4 mm. GrO: granular olfactory bulb, AOn: Anterior olfactory nucleus, Hpc DG: hippocampus dentate gyrus, Hpc CA1: hippocampus CA1, Hpc CA3: hippocampus CA3, SN: Substantia nigra.

The functional coupling of S1P_1_ receptor to G_i/o_ proteins induced by the specific agonist CYM5442 was consistently found to be reduced in the hippocampus, basal ganglia, and rhinencephalon from 3xTg-AD mice. Thus, decreased activity was found in CA1 (3xTg-AD 328 ± 29.2% vs. WT 542 ± 58.7%; *p* < 0.01) and CA3 fields (3xTg-AD 221 ± 11.6% vs. WT 328 ± 33.4%; *p* < 0.01), in the dentate gyrus (3xTg-AD 439 ± 34.9% vs. WT 606 ± 58.8%; *p* < 0.05), substantia nigra (3xTg-AD 544 ± 74.3% vs. WT 855 ± 116.8%; *p* < 0.05), granular olfactory bulb (3xTg-AD 950 ± 65.9% vs. WT 1653 ± 156.9%; *p* < 0.01), and anterior olfactory nucleus (3xTg-AD 804 ± 168.3 vs. WT 1255 ± 107.4%; *p* < 0.05) (Figure 3, Table 3).

### 2.2. Cannabinoid Receptor Density

Cannabinoid receptor density was quantified in 3xTg-AD mice (*n* = 16) and matched WT mice (*n* = 12) using a specific radioligand; the [^3^H]CP55,940. Quantitative densitometry showed that there is an increase in the density of CB_1_ receptors in 3xTg-AD mice at the hippocampal CA1 area (3xTg-AD 284.6 ± 19.8 fmol/mg t.e. vs. WT 193.8 ± 33.8 fmol/mg t.e.; *p* < 0.05) and cingular cortex (3xTg-AD 236.8 ± 17.1 fmol/mg t.e. vs. WT 166.8 ± 27.7 fmol/mg t.e.; *p* < 0.05). The density of CB_1_ receptors was also increased in 3xTg-AD mice at the brain areas with the highest CB_1_ densities, such as the cerebellar gray matter (3xTg-AD 414.3 ± 17.3 fmol/mg t.e. vs. WT 299.5 ± 16.8 fmol/mg t.e.; *p* < 0.01) and the substantia nigra (3xTg-AD 375.9 ± 25.2 fmol/mg t.e. vs. WT 263.8 ± 21.9 fmol/mg t.e.; *p* < 0.01) (Supplementary Material Table S1, Figure S1).

### 2.3. GPCR-Immunoreactivity and Astrocyte Density

**Figure 4:**
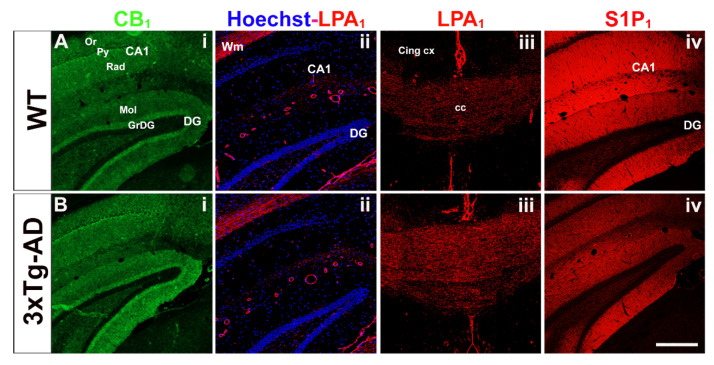
Immunolabeling of CB_1_ (**i**, **green**), LPA_1_ (**ii** and **iii**, **red**), S1P_1_ (**iv, red**) receptors in the hippocampus (**i**, **ii** and **iv**) and corpus callosum (**iii**) from WT (**A**) and 3xTg-AD mice (**B**). Note that CB_1_ and S1P_1_ receptors are distributed in the gray matter, whereas LPA_1_ receptors are mainly expressed in white matter regions. The different hippocampal subfields exhibit specific immunostaining patterns. CA1; CA1 region of hippocampus, DG; dentate gyrus of hippocampus, Or; oriens layer of CA1, Py; pyramidal layer of CA1, Rad; radiatum layer of CA1, Mol; molecular layer of DG, GrDG; granular layer of DG, Wm; white matter, cc; corpus callosum, Cing cx; cingular cortex. Scale bar = 200 µm.

The immunosignaling associated to CB_1_ and S1P_1_ receptors was observed in the gray matter, whereas that associated to LPA_1_ receptor was mainly restricted to white matter regions and only modestly to discrete regions of the gray matter (Figure 4). These observations are in accordance with the observed distribution of the different GPCR functional activity in the autoradiographic studies. In 3xTg-AD mice, S1P_1_ immunoreactivity was found to be decreased in the hippocampus, and LPA_1_ was increased in the corpus callosum, which is in accordance with that observed in the functional coupling to G_i/o_ proteins evoked by the different agonists. Collectively, autoradiographic and immunohistochemical results demonstrate that the changes in the functional activity of these receptors may be directly related to intrinsic variations in the density of these receptors in 3xTg-AD mice.

**Figure 5:**
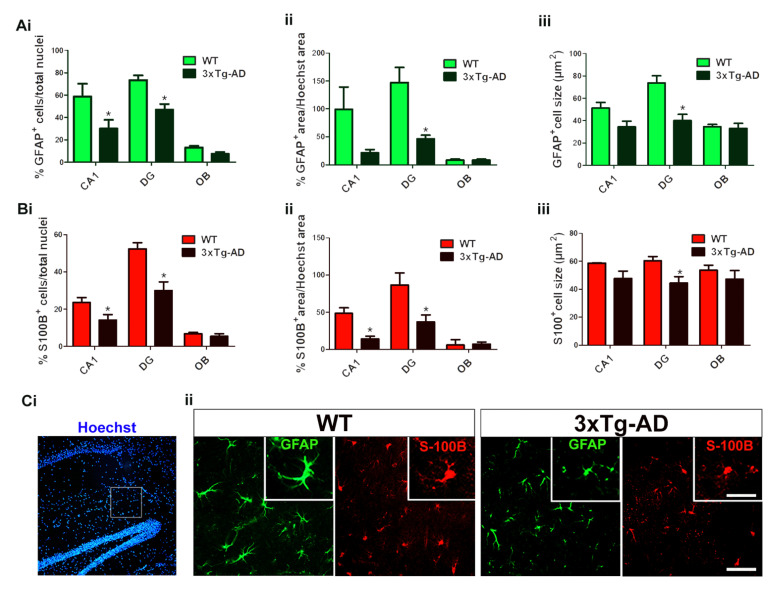
Quantitative analyses of the astrocyte population according to the following; number of cells immunolabeled with GFAP (**A**) or S100B (**B**) over the total cell nuclei (**i**), total area immunolabeled with GFAP or S100B over the total area occupied by cell nuclei (**ii**). Quantitative analyses of the immunolabeled area with GFAP or S100B of individual cells show the astrocyte size (µm^2^) in both genotypes (**iii**). * *p* < 0.05 vs. WT mice. CA1; hippocampus CA1, DG; hippocampus dentate gyrus, OB; olfactory bulb, including granular and anterior. Hoechst staining of nuclei and double labeling of astrocytes in brain tissue from WT and 3xTg-AD revealing particular immunostaining patterns observed with GFAP and S100B, which stain astrocytic processes or cell bodies, respectively (**Ci** and **Cii**). Note the marked decrease in the density of astrocytes as well as their atrophy in the transgenic genotype (3xTg-AD). **Cii** scale bar = 40 µm.

Some of these three neurolipid receptors have also been localized in astrocytes, mainly the S1P_1_ receptor subtype. Therefore, the study of astroglial cells by immunofluorescence was performed in those brain regions which showed marked differences in the functional coupling of the analyzed GPCRs. Two different markers, glial fibrillary acidic protein (GFAP) and S100B, were used to identify astrocytes directly in the tissue. The immunosignal observed by using both markers exhibited different immunostaining patterns showing that astrocytes were mainly distributed in the gray matter. GFAP immunoreactivity clearly delineated the body and the processes of the astrocytes, whereas S100B immunosignal was more restricted and mainly confined to the astrocyte body. The total density of Hoechst-stained nuclei was not modified in transgenic mice. In this sense, both the density of astrocytes and the total area stained with GFAP or S100B were normalized as percentages of total nuclei or total Hoechst-stained area, respectively. Marked changes in density, as well as in cell size, were found in hippocampal CA1 and dentate gyrus fields in 3xTg-AD mice, demonstrating not only a decrease in the population of astrocytes, but also their atrophy or shrinkage. These observations allowed us to clearly differentiate between both genotypes depending on the astroglial-associated immunosignal. However, those differences were not statistically significant in other brain regions such as the granular olfactory bulb (Figure 5).

### 2.4. MALDI-MSI Assay in 3xTg-AD Mice Brain Sections

**Figure 6:**
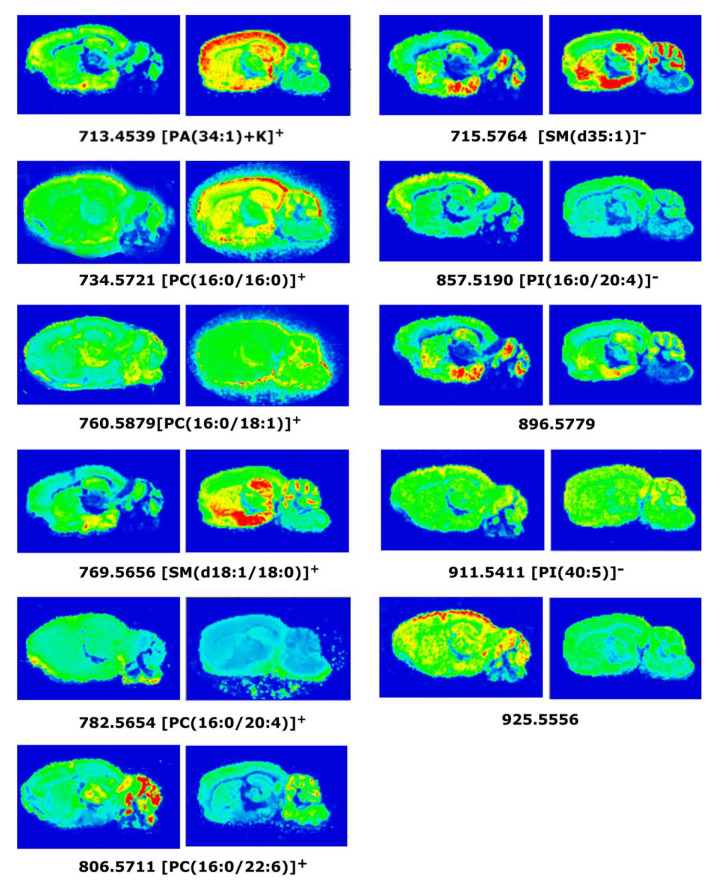
MALDI-MSI images representing different lipid species showing changes in the detection intensities between WT (**left** images) and 3xTg-AD (**right**) mice in sagittal CNS sections.

The most significant differences **in positive ion detection mode** between 3xTg-AD and WT mice were found in the following lipid species: PA[(34:1) + K]^+^; cortex (3xTg-AD 25.0 ± 2.6% vs. WT 15.1 ± 1.5%, *p* < 0.01) and hippocampus (3xTg-AD 25.8 ± 2.9% vs. WT 17.4 ± 1.1%, *p* < 0.05). PC[16:0/16:0]^+^; cortex (3xTg-AD 76.9 ± 7.6% vs. WT 57.2 ± 4.1%, *p*< 0.05) and amygdala (3xTg-AD 75.3 ± 5.9% vs. WT 58.0 ± 0.5%, *p* < 0.05). PC[16:0/18:1]^+^; amygdala (3xTg-AD 78.8 ± 5.1% vs. WT 65.6 ± 1.0%, *p* < 0.05), SM[(d18:1/18:0) + K]^+^; hippocampus (3xTg-AD 35.9 ± 1.4% vs. WT 30.9 ± 0.9%, *p* < 0.05). PC[36:4]^+^; amygdala (3xTg-AD 36.2 ± 2.9% vs. WT 43.9 ± 1.6%, *p* < 0.05), PC[38:6]^+^; and cortex (3xTg-AD 10.5 ± 0.7% vs. WT 15.0 ± 0.2%, *p* < 0.01) (Table 4; Figure 6).

The more significant differences **in negative ion detection mode** were for the following lipid species: SM[d35:1]^−^; cortex (3xTg-AD 37.4 ± 3.7% vs. WT 26.2 ± 1.4%, *p* < 0.05), PI[16:0/20:4]^−^; cortex (3xTg-AD 19.1 ± 0.3% vs. WT 22.7 ± 0.6%, *p* < 0.01) PI[40:5]^−^; cortex (3xTg-AD 8.8 ± 1.7% vs. WT 14.7 ± 0.7%, *p* < 0.01), and one unidentified molecular species at *m*/*z*: 925.5556 (Table 4, Figure 6).

## 3. Discussion

The 3xTg-AD mouse model of AD is an experimental animal model that has been employed for the examination and evaluation of the effects during the development of some of the mechanisms that have been related to genetic familial forms of AD. The aim of the present study was to analyze together the activity of the main receptors for neurolipids present in the central nervous system (CNS): CB_1_, LPA_1_, and S1P_1_ in 3xTg-AD mice at 6 months. The results are discussed in the framework of the lipid composition of the brains in these mice obtained by the MALDI-MSI technique for the in situ analysis, contributing to understand the lipid changes already observed in AD patients and connect these results with the possible adaptations in the activity induced by three different neurolipid-mediated signaling systems: eCB, LPA, and S1P.

### 3.1. Modulation of CB_1_ Receptor Activity

Concerning the cannabinoid system, the activity of CB_1_ receptors, measured as WIN55,212-2-induced [^35^S]GTPγS binding, was lower in the posterior amygdala and layer VI of the motor cortex of 3xTg-AD mice when compared with age-matched WT animals. The cerebral cortex and amygdala belong to the CNS emotional circuitry and contain high levels of CB_1_ receptors [61]. It has been described that the cannabinoid signaling in the prefrontal cortex can modulate the magnitude of neuronal emotional learning plasticity and memory formation through functional inputs from the basolateral amygdala [62]. The amygdala is a region of the temporal lobe that is affected by Aβ and neurofibrillary tangle pathology at early stages of AD. In 3xTg-AD mice, an increase of anxiety and fear related behaviors has been observed and, at the time when Aβ is still localized intraneuronally, some spatial memory deficits appear [63]. In 3xTg-AD mice, Aβ accumulation occurs preferably inside the amygdaloid glutamatergic neurons, where CB_1_ receptors are also located.

Furthermore, we evaluated if the availability and distribution of the CB_1_ receptors in 3xTg-AD mice could account for the above-described results. The analysis of the [^3^H]CP55,940 binding sites in 3xTg-AD mice and age-matched control animals revealed a significant increase of CB_1_ receptor densities in different areas of the 3xTg-AD mice such as the substantia nigra, cerebellum gray matter, dorsal hippocampal CA1 area, and cingular cortex. Studies of CB_1_ receptors in 6-month-old and 10-month-old AβPP/PS1 mice have shown a decrease in CB_1_ receptors in the cortex and hippocampus, respectively [64,65]. Moreover, AβPP/PS1 mice presented higher levels of CB_1_ receptor in the cortex than wild-type mice at 3 months of age [66,67]. Recent studies have reported high levels of CB_1_ mRNA and functional protein in 6-month-old and 7-month-old 3xTg-AD mouse brain in the prefrontal cortex, dorsal hippocampus, and basolateral amygdala [68,69]. The data obtained from the [^3^H]CP55,940 autoradiography did not correlate with the [^35^S]GTPγS binding stimulated by WIN55,212-2, suggesting that receptor density and receptor efficiency can be modulated separately and the contribution of CB_1_ receptors coupled to G_q_ proteins could account for these discrepancies [70]. Previous studies based on human *postmortem* brain samples suggested that CB_1_ receptors could be involved in the pathophysiology of AD [45,46,48,71]. Our research group has observed in patients an increase in CB_1_ density at layer VI of the frontal cortex and different areas of the hippocampus, such as pyramidal layer during the moderate stages of the disease, but having a significant decrease at later stages in the pyramidal layers of the different hippocampal areas and the inner layers of the entorhinal cortex [40].

The decrease of the CB_1_ signaling in 3xTg-AD mice was detected in brain areas innervated by basal forebrain cholinergic neurons, the posterior amygdala, and inner layers of the motor cortex; therefore, a modulation in this pathway on demand of lipid precursors for the further synthesis of eCB could be expected. The synthesis of eCB starts with the release of cell membrane phospholipid precursors such as phosphatidylcholines to further obtain N-arachidonoyl phosphatidylethanolamine or, phosphatidylinositols to further obtain diacylglycerols, in order to synthesize either anandamide or 2-AG, respectively [72]. Interestingly, at least three lipid species which may contain an arachidonic acid (AA) (20:4) moiety were found to be consistently decreased throughout different brain regions including the cortex, the hippocampus, the amygdala, and the cerebellum in the 3xTg-AD mice. MALDI-MSI analyses showed that certain phospholipid species such as PI(16:0/20:4)^−^ and PC(38:6)^+^ which are decreased in the cortex and in the hippocampus, as well as PC(36:4)^+^ species, which is decreased in the amygdala and in the cerebellum, may be being exploited from any membrane pool for this precise biosynthetic process as a physiological adaptation for the observed dysregulation of the CB_1_-mediated signaling.

### 3.2. Modulation of LPA_1_ Receptor Activity

In the present study, an increase in LPA_1_ activity (LPA induced [^35^S]GTPγS binding) was observed in the corpus callosum, motor cortex, hippocampal CA1 area, and striatum of 3xTg-AD mice. Several studies have described that LPA_1_ receptors are expressed in most cell types of the CNS, including neuronal progenitors [73], astrocytes [74], microglia [75,76], and oligodendrocytes [17,77]. We have shown a decrease of microglia and astrocytes in CA1 and DG hippocampal areas of triple transgenic mice, suggesting the increase in LPA_1_ receptor activity in this area could be a compensatory effect due to the decrease of microglia and astrocytes [45]. However, there are other factors that could be related to that increase in LPA_1_ receptor activity, such as myelination disruption. It has been described some degree of myelination disruption in 6-month-old 3xTg-AD mice (early pathological stage) in subregions of hippocampus and entorhinal cortex, together with hyperphosphorylated tau, and a decline of myelin basic protein and 2’,3’-Cyclic-nucleotide 3’-phosphodiesterase expression levels, which are myelin and oligodendrocytes major proteins [78]. Furthermore, in 12-month-old APP_Swe_ mice, myelination defects have also been described in the corpus callosum [79]. Oligodendrocyte myelin sheath integrity is necessary for axon viability and for the maintenance of axonal flow [80]. LPA_1_ receptor has been reported as a novel marker for differentiated oligodendrocytes, suggesting that initiation of LPA_1_ expression may contribute to the myelinating oligodendrocyte phenotype [56,81]. The increase of LPA_1_ receptor activity that we observed at 6-month-old mice (initial stage of the disease) might indicate a neuroprotective action mediated by LPA in response to initial white matter damage. White matter dysfunction seems to appear prior to amyloid or tau pathology in different AD mice models [78,82]. Myelination processes are a vulnerable target contributing to early disease progression. Furthermore, the increased LPA_1_ receptor activity in 3xTg-AD mice at cortex and striatum coincides with the significant increase of PA(34:1) lipid species in the same areas. This increase could be associated with LPA production since LPA is generated on demand from PA by the phospholipase A_2_ enzyme. Interestingly, LPA 18:1 is the most abundant LPA species in brain [83] and PA(34:1) is constituted by oleic acid (18:1). Therefore, the increased LPA_1_ receptor activity could yield to adaptations during the development of 3xTg-AD mice, increasing the demand of LPA endogenous neurotransmitter and increasing the levels of lipid precursors such as PA(34:1) [84].

### 3.3. Modulation of S1P_1_ Receptor Activity

The subtype 1 of sphingosine-phosphate receptors (S1P_1_), a lysophospholipid G_i/o_-coupled GPCR, which is activated by the endogenous neurolipid S1P, was also analyzed. We found an intense S1P_1_ activity in the CNS of both genotypes, but marked reductions in the functional coupling to G_i/o_ proteins in the transgenic mice following the activation with the specific S1P_1_ agonist CYM5442. The S1P_1_ activity was mainly restricted to gray matter, and was even higher than that observed for CB_1_ receptor activity in several brain regions. This S1P_1_ activity may be related to the modulation of neuroinflammatory processes [85]. S1P_1_ receptors are highly expressed in astrocytes and the loss of hippocampal S1P_1_-mediated signaling could be explained due to the loss and/or atrophic processes on astrocytes. This phenomenon had previously been described in the entorhinal cortex from 3xTg-AD mice, and explained as the loss of astrocyte-mediated anti-inflammatory response to Aβ accumulation [86]. Accordingly, the present study shows a clear reduction of S1P_1_ activity in hippocampal areas and a tendency to decrease in the entorhinal cortex from 3xTg-AD mice. The study of astrocytes carried out in the present work using double immunofluorescence images of GFAP/S100B markers clearly demonstrated that both dies are useful to detect and quantify astrocytes. Moreover, the images show particular immunoreactivity patterns that confirm both the decrease of the astrocyte population and their atrophy in the hippocampus. Together, these results are consistent with the idea that following a reduction in the astrocyte-mediated response against the Aβ accumulation, decreased S1P_1_-mediated signaling could contributes to reduce neuroinflammatory responses in this AD mice model. The role of S1P_1_-mediated signaling in AD remains poorly understood, however, the lower expression of sphingosine kinase-1 (S1P-synthesizing enzyme) together with enhanced expression of S1P lyase (S1P-degrading enzyme) lead to the loss of the S1P endogenous ligand pool early in AD [58,59]. Since the use of drugs targeting S1P signaling such as fingolimod (Gilenya^®^, Novartis Pharma AG, Basel, Switzerland), which induces a functional antagonism of S1P_1_ receptors (i.e., reducing S1P_1_-mediated signaling), was approved as immunotherapeutic drug for the treatment of multiple sclerosis, further studies in AD models will contribute to explore the potential of S1P_1_ agonists also for AD treatment. In this sense, the administration of fingolimod to a mouse model overexpressing Aβ led to improve Aβ-associated pathology by attenuating the neuroinflammatory response [85]. It is not clear if the general decrease of S1P_1_ signaling observed in 6-month-old 3xTg-AD mice is a compensatory mechanism to counteract neuroinflammatory events or conversely, is contributing to worsen the pathology, but these evidences point to this neurolipid signaling system as a promising pharmacological target for the treatment of neurodegenerative diseases.

The SM lipid species represent the main phospholipid pool to further synthesize S1P. The increase on the relative abundance of two particular species, SM(18:1/18:0) and SM(d35:1) observed in the hippocampus, where the activity mediated by S1P_1_ receptor was found to be decreased, may indicate a possible cause–effect relationship. The hypoactivity of S1P_1_-mediated signaling in triple transgenic mice could result in the accumulation of these particular SM species due to a lower requirement in the synthesis of S1P. On the other hand, ceramides are well known intermediates in the metabolic pathways of sphingolipids and one would expect to find a decrease in their levels. These ceramides were not detected by MALDI-MSI analysis under the present experimental conditions, preventing a more complete analysis of the metabolic turnover of sphingolipids in this model of AD. These sphingolipids are implicated in the programmed cell death and are directly involved in neurodegeneration, particularly in AD [87]. Interestingly, increased levels of SM(d18:1/18:0) have been found in the hippocampal gray matter as well as in cerebrospinal fluid from AD patients [88,89]. Recently, a relation between ceramide generation and a reduction in mitochondrial ATP release has been reported in astrocytes [90]. In agreement with the present immunofluorescence study, increased levels of specific SM species could be involved in the observed changes in astroglial density and size in 3xTg-AD mice and in relation with S1P signaling. Nevertheless, additional correlational studies measuring the endogenous levels of ceramides and S1P, the enzymatic machinery associated to sphingolipid metabolism, as well as S1P_1_ receptor density will contribute to clarify this issue.

### 3.4. Anatomical Localization of Lipid Species in 3xTg-AD Mice Brain by MALDI-MSI Assay

The field of neurolipidomics tries to understand how dynamic changes in membrane lipid composition are contributing to regulate brain cell function. Previous studies have indicated that lipid molecules play a relevant role in AD, and some of these lipids have frequently been reported at abnormal concentrations in AD tissue [35,91,92,93]. Although several studies have been performed with AD transgenic models showing lipid impairments [94,95,96,97,98], the present study is pioneer in achieving anatomical localization of lipid species in 3xTg-AD mice brain by MALDI-MSI assay. We have observed modifications on PC, PA, SM, and PI intensities in different brain areas. Moreover, the modulations of PC and PI species depend on their fatty acid composition, i.e., decrease of polyunsaturated fatty acid (PUFA) phospholipids and increase of phospholipid species containing saturated fatty acids (SFA). Similar results have been reported in serum and tissue of AD patients [31,32,33,35] and also in another AD transgenic mice model [97,99]. The decrease in phospholipids containing PUFA could be related to impairment of the cell membranes during AD pathogenesis. Some morphological and neurofunctional damages have been found to correlate with PUFA declines, including swollen astrocytes, deformed nerve cell nuclei, reduced acetylcholine release, and modifications on the fluidity, structure, and permeability of the cell membranes [100,101,102]. Furthermore, the increase in the PA(34:1) species in 3xTg-AD mice brain, could be related with an increase in total phospholipase D activity that has been reported in AD brain homogenates, using an in vitro enzymatic assay [103]. In addition, decrease in PC species have been described as possible plasma biomarkers for AD, even in patients before the onset of the disease [104]. In our study, we have found a decrease in one of that PC species, that could be induced by upregulation of PLA_2_ enzyme in AD [105,106]. In contrast, we have not found a decrease of glucosylceramides or sulfatides as have been reported in the APP/PS1 and APP/tau transgenic mice lineages or even in patients at the first stages of the disease [97,98,107].

In summary, the modulation of the main CNS receptors of the LPA, eCB, and S1P neurolipid systems analyzed in the triple transgenic model of AD suggests a neuroprotective adaptation during the development of these mice. The cannabinoid activity improving or maintaining the neurotransmission, LPA activity trying to relieve the myelination dysfunction in the axons, and S1P_1_ activity attenuating astrocyte-mediated neuroinflammatory response [108]. In addition, the observed changes on lipid species in the 3xTg-AD mice in specific brain regions suggest a similar modulation in the cases of familial AD patients, which are covered by this mouse model.

Further studies will help us to shed light on the relevance of the observed modifications and if they are indicating primary effects or are a physiological outcome of the neurodegeneration. The complexity of the AD biochemistry in the brain is probably a consequence of multiple causes that are converging in the observed clinical manifestations that include the progressive dementia. The research on neurolipid signaling and their control on the lipid homeostasis and modulation of other neurotransmitter systems has been limited by the techniques used to anatomically identify the super-specialization on lipid species in the brain, which reaches the highest levels in the human cortex. The MSI used in the present study combined with other neuroanatomical methods will open new perspectives in our ultimate goal of understanding the integration of energetic, structural, and signaling functions mediated by lipid molecules in the brain that will contribute to develop specific and effective treatments for neuropsychiatric and neurodegenerative diseases, including AD.

## 4. Materials and Methods

### 4.1. Chemicals

[^35^S]GTPγS (initial specific activity 1250 Ci/mmol) and [^3^H]CP55,940 (initial specific activity 144 Ci/mmol) were purchased from Perkin Elmer (Boston, MA, USA), Oleoyl-L-α-lysophosphatidic acid sodium salt was obtained from Sigma-Aldrich (St. Louis, MO, USA), WIN55,212-2 was purchased from Tocris, 2-mercaptobenzothiazole (MBT) was acquired from Sigma-Aldrich (St. Louis, MO, USA). The [^14^C]-microscales used as standards in the autoradiographic experiments were purchased from Amersham Biosciences (St. Louis, MO, USA). Moreover, DL-dithiothreitol (DTT), guanosine-5′-diphosphate (GDP) and guanosine-5′-o-3-trisphosphate were provided from Sigma (St. Louis, MO, USA), the β-sensitive films Kodak Biomax MR were supplied from Sigma (St. Louis, MO, USA). Finally, for the preparation of the incubation buffers, the treatment of slides, re-crystallization of the matrix and films developing, several different compounds supplied from different companies were used, and all the compounds were of the highest commercially available quality for the necessity of the neurochemical studies.

### 4.2. Animals and Tissue Preparation

Triple transgenic mice (3xTg-AD) were obtained from Department of Psychiatry and Forensic Medicine, Universitat Autònoma de Barcelona, Barcelona, Spain, in collaboration with Dr. Lydia Giménez-Llort. 3xTg-AD mice harboring PS1_M146V_, APP_Swe_ and tau_P301L_ transgenes were genetically engineered at the University of California Irvine, as previously described [19]. Briefly, two independent transgenes (encoding human APP_Swe_ and human tau_P301L_, both under control of the mouse Thy1.2 regulatory element) were co-injected into single-cell embryos harvested from homozygous mutant PS1 _M146_V knock-in (PS1KI) mice.

Six-month-old male 3xTg-AD mice (*n* = 26) and WT mice with the same background but without genetic modifications (*n* = 22) were used. The breeding program was established at the Universitat Autònoma de Barcelona. All the animals were housed and maintained under standard laboratory conditions (12 h light:dark, cycle starting light at 8:00 am, food and water available *ad libitum*, 22 ± 2 °C, 50–60% humidity). Animals were transferred to the animal department of UPV/EHU, with the same standard housing conditions, one month before the experimental procedures. All procedures were performed in accordance with European animal research laws (European Communities Council Directives 86/609/EEC, 98/81/CEE and 2003/65/CE; Commission Recommendation 2007/526/EC) and the Spanish National Guidelines for Animal Experimentation and the Use of Genetically Modified Organisms (Real Decreto 1205/2005 and 178/2004; Ley 32/2007 and 9/2003). Experimental protocols were approved by the Local Ethical Committee for Animal Research at the University of the Basque Country (CEIAB/52&54/2018/Rodriguez Puertas).

### 4.3. Tissue Preparation

Mice were deeply anesthetized with ketamine/xylazine (90/10 mg kg^−1^; i.p.).

Fresh tissue. The brain samples were quickly removed by dissection, fresh frozen, and kept at −80 °C. Later, the brains were cut on a Microm HM550 cryostat (Thermo Fisher Scientific, Whaltham, MA, USA) to obtain 20 µm sections that were mounted onto gelatin-coated slides and these were stored at −20 °C until used.

Fixed tissue. Three animals from each genotype were transcardially perfused via the ascending aorta with 50 mL warm (37 °C), calcium-free Tyrode’s solution (0.15 M NaCl, 5 mM KCl, 1.5 mM MgCl_2_, 1 mM MgSO_4_, 1.5 mM NaH_2_PO_4_, 5.5 mM Glucose, 25 mM NaHCO_3_; pH 7.4), 0.5% heparinized, followed by 4% paraformaldehyde and 3% picric acid in 0.1M PB (4 °C) (100 mL/100 g b.w.). The brains were subsequently removed and post-fixed in the same fixative solution for 90 min at 4 °C, followed by immersion in 20% sucrose in PB cryoprotective solution overnight at 4 °C. Then, the tissue was frozen by immersion in isopentane and kept at −80 °C. The brains were coronally cut at 10 µm sections using a Microm HM550 cryostat (Thermo Fisher Scientific, Whaltham, MA, USA) equipped with a freezing-sliding microtome at −25 °C and mounted onto gelatin-coated slides and stored at −25 °C until used.

### 4.4. [^35^S]GTPγS Binding Assay

The tissue sections were air-dried for 15 min. Then, slides containing the sections were washed in a HEPES based buffer containing 50 mM HEPES, 100 mM NaCl, 3 mM MgCl_2_ and 0.2 mM EGTA, 0.5% bovine serum albumin (BSA) at pH 7.4, for 30 min at 30 °C in a water bath. The pre-incubation was repeated a second time in new buffer to ensure the washing of endogenous GPCR ligands. In a second step, the slides were incubated for 2 h at 30 °C in a solution containing 2 mM guanosine diphosphate (GDP), 1 mM DL-dithiothreitol (DTT), adenosine deaminase (3 u/L) and 0.04 nM [^35^S]GTPγS. The agonist-stimulated binding was measured under the same conditions but in the presence of the specific GPCR agonists: LPA (10^−5^ M), WIN55,212-2 (10^−5^ M) and CYM5442 (10^−5^ M). Ki16425 (10^−5^ M) was used together with LPA, AM251 (10^−5^ M) with WIN55,212-2, and W146 (10^−5^ M) with CYM5442 as respective antagonists to validate that the assays were specific of the receptor subtype. Non-specific binding was determined in the presence of 10 μM of non-labelled GTPγS. Sections were washed twice in an ice-cold HEPES buffer 50 mM (pH 7.4), dipped in distilled water, and air-dried. Sections were exposed to Kodak Biomax MR films (Sigma, St. Louis, MO, USA) together with ^14^C standards for 48 h at 4 °C.

### 4.5. Quantitative Image Analysis of Film Autoradiograms

Films were scanned and quantified by transforming the optical densities into nCi/g tissue equivalent (nCi/g t.e.) and percentage of stimulation over the basal (%) was calculated using an image analysis system (NIH-IMAGE, Bethesda, MA, USA). (U.S National Institutes of Health, http://rsb.info.nih.gov/nih-image/). This software defines the optical density of an anatomical area from 0 (white) to 256 (black). The [^14^C] radioactive standards that were co-exposed with the slides were used to calibrate the optical densities with the level of radioactivity labeled to the sections. Experimental data were analyzed by using the computer programs GraphPad Prism (v. 5.0, Graph Pad) and Microsoft office Excel 2007. Data were expressed as the mean values ± SEM. Differences between regions were analyzed by unpaired two-tailed Student’s *t* test.

### 4.6. Immunofluorescence Studies

Prior to staining procedures, sections were air dried for 20 min, extensively rinsed with 0.1M phosphate buffer (PBS, pH 7.4) and blocked with 4% normal goat serum in PBS for 2 h at room temperature. To detect astrocytes, brain tissue sections were incubated (4 °C, overnight) with a mixture of rabbit polyclonal anti S100B (1:800) (Millipore, Temecula, CA, USA) and mouse monoclonal anti GFAP (1:1000) (Millipore, Temecula, CA, USA) to detect both immature and more mature developmental stages. To detect S1P_1_ and LPA_1_ receptors, mouse monoclonal anti S1P_1_ (1:400) (Millipore, Temecula, CA, USA) and rabbit polyclonal anti LPA_1_ (1:300) (Thermo Fisher Scientific, Whaltham, MA, USA) antibodies were used in consecutive brain sections. Primary antibodies were diluted in Triton X-100 (0.3%) in PBS with 5% BSA. The sections were then rinsed with PBS followed by incubation with carbocyanine (Cy3)-conjugated donkey anti-rabbit IgG (1:250), (Cy3)-conjugated donkey anti-mouse IgG (1:250) (Jackson Immunoresearch, PA) and FITC-goat anti-mouse (1:80) (Jackson Immunoresearch Laboratories, Inc., West Grove, PA, USA) for 30 min at 37 °C in the darkness. Secondary antibodies were diluted in Triton X-100 (0.3%) in PBS. To label CB_1_ receptors, the primary rabbit antiserum against the CB_1_ receptor, PA1-743, (Affinity BioReagents, CO, USA) was diluted [1:500] in TBS (0.1 M Tris, 0.15 M NaCl, pH 7.4) containing 0.5% milk powder. The tyramide signal amplification method was used to amplify the signal associated with the CB_1_ receptor antiserum. Briefly, sections were washed for 30 min in TNT buffer (0.05% Tween 20 in TBS, pH 7.4) and blocked in TNB solution (10 mL TNT buffer, 0.05 g blocking reagent, (DuPont NEN, Boston, MA, USA)) for 1 h at room temperature. Later, the sections were incubated with horseradish peroxidase-conjugated goat anti-rabbit secondary antibody (Perkin Elmer, Whaltham, MA, USA) for 1 h followed by tyramide fluorescein-based amplification process in complete darkness for 10 min at room temperature. Sections were extensively rinsed in TBS. Then, in order to stain nuclei, all sections were washed for 30 min by immersion in PBS and incubated with bisbenzimide H33258 (Hoechst [1:10^6^]) for 15 min at room temperature. Finally, sections were extensively rinsed with PBS and mounted with p-phenylendiamine-glycerol (0.1%) in PBS for immunofluorescence.

### 4.7. Quantitative Analyses of Astrocytes

Sections were inspected and immunofluorescence images from WT (*n* = 3) and 3xTg-AD (*n* = 3) were used to quantify the astrocyte density; 400-fold magnification photomicrographs (SPOT Flex Shifting Pixel CCD imaging camera) were acquired on an Axioskop 2 Plus epifluorescence microscope (Carl Zeiss, Oberkochen, Germany) in both hemispheres under the same microscopic conditions. Using Image J software (NIH, Bethesda, MD, USA), images were converted to a binary mode and different processes were performed in order to identify single astrocytes and nuclei by applying the watershed option. The total number of astrocytes and nuclei (N/mm^2^) were quantified, and the total area (in pixels) stained by astrocytes (S100B^+^ or GFAP^+^-immunoreactivity) or nuclei (Hoechst staining) and each astrocyte and nuclei stained area (size in pixels) were calculated in each image. Hoechst stained nuclei were used to normalize the number of GFAP or S100B positive cells in each image (% of astrocytes of total nuclei). Hoechst stained area was used to normalize the GFAP and/or S100B positive area in each image (% of GFAP or S100B immunopositive area of total Hoechst-stained area).

### 4.8. Sample Preparation for MALDI-MSI

The original lipid composition and anatomical characteristics of the tissue must be preserved throughout the sample-preparation process [109]. The brains were cut on a Microm HM550 cryostat to obtain 20-µm sections and stored at −20 °C until the moment of use.

Once the initial tissue preparation steps had been completed, the chemical matrix was deposited on the tissue surface prior to analysis by sublimation. For tissue sections mounted on glass slides, sublimation was performed using 300 mg of mercaptobenzothiazole (MBT), by controlling the deposition time and temperature (30 min at 140 °C), making it possible to control the thickness of the matrix layer and optimize the s/n ratio of the mass spectra, avoiding lipid migration thanks to the lack of solvent. Finally, a re-crystallization of the sample was performed, using a normal glass Petri plate (100 mm diameter × 15 mm depth (Thermo Fisher Scientific, Whaltham, MA, USA)) as following. Thus, 1 mL of methanol (99%) was deposited onto a piece of paper previously placed in the bottom of the Petri plate in order to create a vapor atmosphere for the re-crystallization process on a hot plate (1 min at 40 °C). This step allowed us to achieve a higher intensity in the detection of the peaks [110].

### 4.9. Mass Spectrometer

A MALDI LTQ-XL-Orbitrap (Thermo Fisher, San Jose, CA) equipped with a nitrogen laser (λ = 337 nm, rep. rate = 60 Hz, elliptical spot size = 80 × 120 μm^2^) was used for mass analysis. Thermo’s ImageQuest^TM^ 1.0.1 and Xcalibur^TM^ 3.1 software were used for MALDI-MSI data acquisition. The images were acquired in both negative and positive ion mode. The positive ion range was 500–1000 Da, with 10 laser shots per pixel at a laser fluence of 15 μJ. The negative ion range was 400–1100 Da, with 10 laser shots per pixel at laser fluence of 15 μJ. The target plate stepping distance was set to 150 μm for both the x- and y-axes by the MSI image acquisition software. The mass resolution was 100,000 in both positive and negative ion mode. The data were normalized using the total ion current to avoid the displacement in masses along the tissue caused by irregularities on the surface or other experimental artifacts.

### 4.10. Image and Spectra Analysis for MALDI-MSI

The MALDI images were generated using the ImageQuest software (Thermo Scientific, San Jose, CA, USA). With this software, a *m*/*z* range is plotted for signal intensity for each pixel (mass spectrum) across a given area (tissue section). The quality of the images was improved during the image creation process by selecting the *m*/*z* range of interest and doing a normalization as a ratio of total ion current (TIC) for each mass spectrum. Different regions of interest (ROI) were analyzed including hippocampus, cortex, amygdala, cerebellum, and striatum. The spectra intensity was further normalized as a ratio of the peak or *m*/*z* value with the highest intensity, PC[(34 + 1) + K]^+^ in positive ion mode and PI[18:0/20:4]^−^ in negative ion mode and the average was calculated using the OriginPro 8 software. The most intense peak was considered the 100% and the intensity of the rest of the peaks was calculated as a percentage. The two-tailed unpaired Student’s *t*-test was used for the comparison of two groups. The results were considered significant when *p* ≤ 0.05.

### 4.11. Peak Assignment

The assignment of the *m*/*z* values to specific molecules is complex in this type of studies, usually containing a large number of lipids that share similar masses. Therefore, the assignment of lipid species was facilitated using databases such as Lipid MAPS (http://www.lipidmaps.org/ accessed on 9 November 2021), and different reported articles. A 5 ppm mass accuracy was used as the tolerance window. The glycerolipid species numbers (x:y) denote the total lengths and the number of double bonds of the acyl chains, while the sphingolipid and sulfatide species numbers correspond to the length and number of double bonds of the acyl chain added to those of the attached sphing-4-enine (d18:1) or sphinganine (d18:0) base.

## Figures and Tables

**Table 1:** [^35^S]GTPγS basal binding in different areas of WT and 3xTg-AD mice brain (nCi/g t.e) and induced by WIN55,212-2 (10 µM) expressed in percentage of stimulation over the basal. *n* (WT) = 12; *n* (3xTg-AD) = 16. Data are mean ± SEM values.

	Basal Binding (nCi/g t.e.)		WIN55,212-2 Stimulation (%)	
Brain Region	WT	3xTg-AD	WT	3xTg-AD
Amygdala				
Anterior	234.9 ± 18.3	218.2 ± 20.9	204.5 ± 30.1	185.8 ± 37.7
Posterior	216.6 ± 14.6	266.4 ± 23.5	295.5 ± 41.7	112.8 ± 28.9 **
Internal capsule	143.1 ± 19.0	120.8 ± 9.8	52.9 ± 12.5	36.2 ± 11.1
Striatum	178.8 ± 8.4	187.4 ± 12.8	250.2 ± 27.7	226.1 ± 26.4
Cerebellum				
White matter	60.2 ± 6.5	51.7 ± 5.7	66.1 ± 34.8	93.2 ± 19.6
Gray matter	54.7 ± 4.7	66.7 ± 7.3	814.8 ± 56.3	658.2 ± 49.2
Cortex				
Cingular	164.2 ± 13.0	166.0 ± 12.4	308.4 ± 45.5	267.3 ± 38.2
Motor Layers I-VI	152.4 ± 7.9	178.4 ± 13.1	267.3 ± 25.6	228.6 ± 29.7
Layer VI	175.3 ± 55.4	190.8 ± 52.8	435.4 ± 58.2	238.4 ± 22.9 **
Corpus callosum	146.6 ± 11.8	127.2 ± 10.2	72.1 ± 13.1	84.6 ± 14.5
Globus pallidus	200.6 ± 12.3	204.7 ± 17.7	870.2 ± 69.4	872.5 ± 52.2
Hippocampus				
CA1	132.3 ± 10.8	143.4 ± 9.9	314.8 ± 47.1	238.4 ± 27.1
Dentate gyrus	121.1 ± 15.4	143.5 ± 8.8	209.8 ± 38.9	202.5 ± 21.5
Hypothalamus	217.6 ± 16.6	243.3 ± 22.8	162.5 ± 20.8	169.3 ± 16.3
Thalamic nuclei				
Anteroventral	113.7 ± 11.7	138.6 ± 10.2	128.5 ± 35.4	86.1 ± 30.3
Thalamus	89.8 ± 7.8	112.7 ± 9.9	109.3 ± 29.9	92.5 ± 15.6
Basal Nucleus	215.6 ± 14.7	227.3 ± 15.9	271.3 ± 39.1	274.9 ± 39.9
Substantia Nigra	190.1 ± 16.9	172.1 ± 8.1	1062.7 ± 79.8	979.3 ± 65.7

The *p* values were calculated by two-tailed Student’s *t* test ** *p* ≤ 0.01.

**Table 2:** [^35^S]GTPγS induced by LPA (10 µM) in different areas of WT and 3xTg-AD mice brain expressed in percentage of stimulation over the basal. *n* (WT) = 12; *n* (3xTg-AD) = 16. Data are mean ± SEM values.

	LPA Stimulation (%)	
Brain Region	WT	3xTg-AD
Amygdala		
Anterior	20.5 ± 11.7	13.0 ± 10.3
Posterior	28.8 ± 12.8	8.1 ± 15.9
Internal capsule	30.2 ± 9.6	32.8 ± 6.2
Striatum	3.3 ± 7.2	23.1 ± 3.8 *
Cerebellum		
White matter	76.6 ± 18.4	111.3 ± 19.7
Gray matter	62.0 ± 15.7	82.9 ± 19.7
Cortex		
Cingular	13.2 ± 7.2	20.8 ± 6.4
Motor	6.2 ± 12.4	26.7 ± 7.6 *
Corpus Callosum	90.8 ± 12.3	189.6 ± 17.4 *
Globus pallidus	22.1 ± 9.3	29.8 ± 6.9
Hippocampus		
CA1	−18.7 ± 7.8	22.7 ± 4.4 *
Dentate gyrus	54.7 ± 11.0	49.5 ± 23.2
Hypothalamus	34.5 ± 19.9	22.6 ± 10.2
Thalamic nuclei		
Anteroventral	22.6 ± 14.2	20.6 ± 8.2
Thalamus	20.6 ± 13.2	24.6 ± 11.1
Basal Nucleus	22.9 ± 10.7	39.8 ± 8.5
Substantia Nigra	23.1 ± 13.7	25.4 ± 7.8

The *p* values were calculated by two-tailed Student’s *t* test * *p* ≤ 0.05.

**Table 3:** [^35^S]GTPγS induced by CYM5442 (10 µM) in different areas of WT and 3xTg-AD mice brain expressed in percentage of stimulation over the basal. *n* (WT) = 7; *n* (3xTg-AD) = 7. Data are mean ± SEM values.

	**CYM5442 Stimulation (%)**	
**Brain Region**	**WT**	**3xTg-AD**
Amygdala		
Anterior	487 ± 91.4	515 ± 91.7
Posterior	334 ± 33.9	397 ± 49.5
Internal capsule	193 ± 45.0	131 ± 16.0
Striatum	446 ± 58.9	375 ± 29.1
Cerebellum		
White matter	183 ± 37.1	166 ± 19.5
Gray matter	329 ± 41.9	343 ± 52.3
Cortex		
Cingular	789 ± 131.0	997 ± 173.0
Motor	690 ± 94.0	677 ± 79.0
Entorhinal	542 ± 104.0	387 ± 52.0
Frontal	475 ± 46.3	483 ± 56.0
Corpus callosum	243 ± 41.0	196 ± 29.0
Globus pallidus	468 ± 61.9	365 ± 41.3
Hippocampus		
CA1	542 ± 58.7	328 ± 29.2 **
CA3	328 ± 33.4	221 ± 11.6 **
Dentate gyrus	606 ± 58.8	439 ± 34.9 *
Hypothalamus	178 ± 50.16	188 ± 37.2
Thalamic nuclei		
Anteroventral	177 ± 35.2	204 ± 62.8
Thalamus	188 ± 48.7	209 ± 50.1
Basal Nucleus	448 ± 46.2	373 ± 23.9
Substantia Nigra	855 ± 116.8	544 ± 74.3 *
Granular olfactory bulb	1653 ± 156.9	950 ± 65.9 **
Anterior olfactory Nucleus	1255 ± 107.4	804 ± 168.3 *

The *p* values were calculated by two-tailed Student’s *t* test * *p* ≤ 0.05, ** *p* ≤ 0.01.

**Table 4:** Percentage of the intensity of molecular lipid species in positive and negative mode in sagittal mice sections from WT (*n* = 6) compared to the 3xTg-AD (*n* = 6), as revealed by MALDI-MSI. Data are mean ± SEM values.

		Cortex		Hippocampus		Striatum		Amygdala		Cerebellum	
Assignment	*m*/*z*	WT	3xTg-AD	WT	3xTg-AD	WT	3xTg-AD	WT	3xTg-AD	WT	3xTg-AD
PA(34:1)+K^+^	713.4535	15.1 ± 1.5	25.0 ± 2.6 **	17.4 ± 1.1	25.8 ± 2.9 *	13.7 ± 0.9	20.0 ± 2.4 *	22.7 ± 1.8	25.7 ± 3.3	14.1 ± 2.4	15.3 ± 2.2
PC(16:0/16:0)^+^	734.5721	57.2 ± 4.1	76.9 ± 7.6 *	70.1 ± 4.3	71.8 ± 4.9	69.8 ± 3.4	69.2 ± 3.5	58.0 ± 0.5	75.3 ± 5.9 *	55.5 ± 4.3	70.9 ± 3.1 *
PC(16:0/18:1)^+^	760.5658	82.3 ± 5.7	83.4 ± 4.8	86.1 ± 4.5	87.2 ± 4.8	88.7 ± 5.7	90.1 ± 6.9	65.6 ± 1.0	78.8 ± 5.1 *	96.0 ± 2.1	96.1 ± 2.1
SM(d18:1/18:0)+K^+^	769.5656	38.0 ± 3.5	36.1 ± 1.5	30.9 ± 0.9	35.9 ± 1.4 *	23.3 ± 2.5	25.1 ± 1.6	34.8 ± 1.8	41.0 ± 1.5 *	25.1 ± 3.0	26.5 ± 2.0
PC(36:4)^+^	782.5654	33.1 ± 2.9	30.7 ± 2.1	37.3 ± 1.7	35.4 ± 1.1	34.6 ± 1.1	32.7 ± 1.3	43.9 ± 1.6	36.2 ± 2.9 *	29.8 ± 0.8	24.9 ± 1.1 **
PC(38:6)^+^	806.5711	15.0 ± 0.2	10.5 ± 0.7 **	9.8 ± 0.9	9.3 ± 0.5	11.3 ± 0.8	10.7 ± 1.3	5.3 ± 0.8	6.4 ± 0.8	19.9 ± 1.1	13.1 ± 1.2 **
SM(d35:1)^−^	715.5764	26.2 ± 1.4	37.4 ± 3.7 *	50.2 ± 2.8	65.0 ± 5.9 *	35.4 ± 2.1	38.1 ± 2.9	55.5 ± 2.8	67.5 ± 3.1 *	41.2 ± 5.3	48.3 ± 6.0
PI(16:0/20:4)^−^	857.5190	22.7 ± 0.6	19.1 ± 0.3 **	14.5 ± 0.5	11.1 ± 0.3 **	12.1 ± 0.2	11.6 ± 0.2	10.5 ± 0.2	9.1 ± 0.6	10.8 ± 0.6	9.7 ± 1.1
CPI(40:2)+MBT	896.5779	11.2 ± 1.5	12.7 ± 2.8	23.8 ± 1.1	23.9 ± 2.1	15.3 ± 1.1	19.7 ± 3.1	25.5 ± 2.1	17.2 ± 2.0 *	20.9 ± 3.3	17.6 ± 2.5
PI(40:5)^−^	911.5411	14.7 ± 0.7	8.8 ± 1.7 **	11.3 ± 0.7	9.9 ± 1.2	10.2 ± 0.6	10.3 ± 1.1	9.6 ± 1.1	8.7 ± 1.9	10.1 ± 0.8	8.2 ± 1.6
	925.5556	14.8 ± 1.7	6.3 ± 2.8 *	11.8 ± 0.9	3.7 ± 2.1 **	9.8 ± 1.5	7.5 ± 2.7	11.9 ± 0.9	5.7 ± 2.8 *	10.5 ± 1.3	8.1 ± 2.7

The *p* values were calculated by two-tailed Student’s *t* test * *p* ≤ 0.05, ** *p* ≤ 0.01. PA: phosphatidic acid, PC: phosphatidylcholine, SM: sphingomyelin, PI: phosphoinositol, CPI: ceramide phosphoinositol.

## Supplementary Materials

The following are available online at https://www.mdpi.com/article/10.3390/ijms222212256/s1.
