# Supplementary material for: Modulation of Neurolipid Signaling and Specific Lipid Species in the Triple Transgenic Mouse Model of Alzheimer’s Disease
Source: Int J Mol Sci. 2021 Nov 12;22(22):12256. doi: 10.3390/ijms222212256 (PMC8620566; doi:10.3390/ijms222212256)
Supplement: Supplementary file 1 [file ijms-22-12256-s001.zip › ijms-1451298-supplementary.pdf]

SUPPLEMENTARY METHODS AND DATA

González de San Román et al.: MODULATION OF NEUROLIPID SIGNALING AND SPECIFIC LIPID SPECIES IN THE TRIPLE TRANSGENIC MOUSE MODEL OF ALZHEIMER´S DISEASE

Cannabinoid receptor autoradiography

For cannabinoid receptor autoradiography, 20 μm thickness consecutive slices were incubated (30 min, 22 ± 2°C) in a 50 mM Tris-HCl buffer containing 1% BSA, pH 7.4. Later, two consecutive sections were incubated for 2h at 37ºC with 3 nM of [^{3}H]CP55940 in the same buffer, pH 7.4, containing 1% BSA. Non-specific binding was determined incubating other consecutive sections in the presence of 10 μM of AM251 (Tocris, USA). Sections were rinsed twice in ice-cold Tris-HCl 50 mM buffer supplemented with 0,5% BSA (pH 7.4) to remove unbound ligand, and then slides were dipped in distilled water (4ºC). After drying, autoradiograms were generated by exposing the tissues for 21 days at 4ºC to β-radiation sensitive films (Kodak Biomax MR, Sigma), together with [^{3}H]-microscales used as standards in the autoradiographic experiments, which were purchased from Amersham Biosciences (St. Louis, USA), to calibrate the optical densities to fmol/mg tissue equivalent (fmol/mg t.e.), as mean ± SEM, as described below. The [^{3}H] radioactive standards that were co-exposed with the slides, were used to calibrate the optical densities with the level of radioactivity labeled to the sections. Experimental data were analyzed by using the computer programs GraphPad Prism (v. 5.0, Graph Pad) and Microsoft office Excel 2007. Data were expressed as the mean ± SEM. Differences between regions were analyzed by unpaired two-tailed Student's *t test*.

Table S1. Autoradiographic densities for the specific binding of [^{3}H]CP55.940 in WT and 3xTg-AD mice (fmol/mg). n (WT) = 12; n (3xTg-AD) = 16. Data are mean ± SEM values.

	Specific binding [^{3}H]CP 55.940 (fmol/mg)	
**Brain region**	**WT**	**3xTg-AD**
Amygdala		
Anterior	71.3 ± 6.3	88.1 ± 10.9
Posterior	87.6 ± 9.5	109.7 ± 14.3
Internal capsule	85.0 ± 14.4	68.6 ± 15.4
Striatum	90.2 ± 21.9	85.9 ± 17.9
Cerebellum		
White matter	35.0 ± 11.6	10.6 ± 7.6
Gray matter	299.5 ± 16.8	414.6 ± 17.3**
Cortex		
Cingulate	166.8 ± 27.7	236.8 ± 17.1*
Motor	166.2 ± 7.1	129.5 ± 23.6
Corpus Callosum	13.2 ± 5.9	19.9 ± 5.5
Globus pallidus	202.4 ± 17.2	217.6 ± 24.5
Hippocampus		
CA1	193.8 ± 33.8	284.6 ± 19.5*
Dentate gyrus	143.2 ± 24.4	159.2 ± 31.5
Hypothalamus	46.5 ± 6.8	56.4 ± 8.6
Thalamic nuclei		
AVVL	12.6 ± 6.9	20.8 ± 5.5
Thalamus	21.0 ± 6.0	18.4 ± 3.7
Basal Nucleus	61.6 ± 2.2	87.1 ± 12.2
Substantia Nigra	263.8 ± 21.9	375.9 ± 25.2**

The *p* values were calculated by two-tailed Student's *t test* *$p \leq 0.05$, **$p \leq 0.01$.

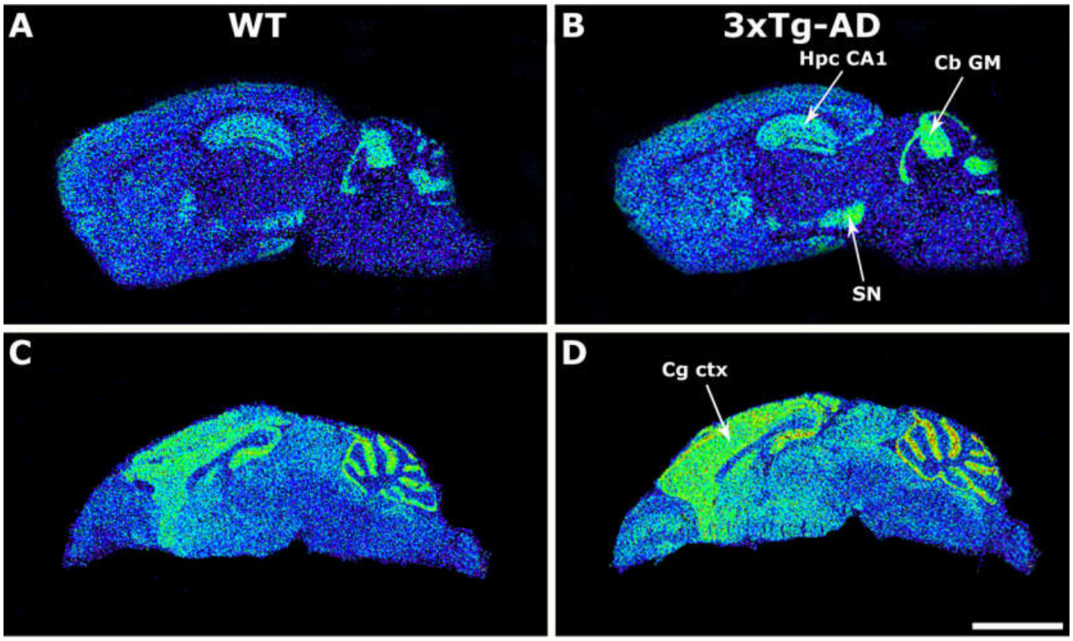

Figure S1. Representative autoradiograms of WT (**A**,**C**) and 3xTg-AD (**B**,**D**) mouse in sagittal sections that show specific binding of [^{3}H]CP55.940. An increase in the density of CB_1 receptors was observed in 3xTg-AD mice at brain areas where intracellular Aβ has been built up, such as hippocampal CA1 area and cingulate cortex. The density of CB_1 receptors was also increased in 3xTg-AD mice at the brains areas with the highest CB_1 densities, such as cerebellar gray matter and substantia nigra. Scale bar = 3 mm. cc; corpus callosum, cb GM; cerebellum gray matter, Hpc CA1; hippocampus CA1, Cg ctx; cingulate cortex.
